# Doping Prevalence among U.S. Elite Athletes Subject to Drug Testing under the World Anti-Doping Code

**DOI:** 10.1186/s40798-024-00721-9

**Published:** 2024-05-20

**Authors:** Ann Kearns Davoren, Kelly Rulison, Jeff Milroy, Pauline Grist, Matthew Fedoruk, Laura Lewis, David Wyrick

**Affiliations:** 1 Prevention Strategies, Greensboro, NC USA; 2 The University of North Carolina Greensboro, Greensboro, NC USA; 3 U.S. Anti-Doping Agency (USADA), Colorado Springs, CO USA

**Keywords:** Doping prevalence, Performance enhancing drugs, Athletes

## Abstract

**Background:**

Determining the prevalence of doping within an elite athlete population is challenging due to the extreme sensitivity of the topic; however, understanding true doping prevalence is important when designing anti-doping programs and measuring their effectiveness. The objective of this study was to estimate the prevalence of doping among Olympic, Paralympic, World, and National-level competitive athletes in the United States subject to the World Anti-Doping Code. All athletes who were subject to the U.S. Anti-Doping Agency’s Protocol for Olympic and Paralympic Movement Testing, a World Anti-Doping Code (“Code”)-compliant anti-doping program, were invited to complete a web-delivered survey. Using a direct questioning approach, the survey items asked athletes whether they had used each specific category of banned substance / method on the World Anti-Doping Agency’s Prohibited List. Multiple strategies to encourage honest reporting (e.g., protecting anonymity by collecting minimal demographic information; using an outside organization to administer the survey) and to detect inconsistent responses were used.

**Results:**

Depending on the method of calculation, 6.5–9.2% of the 1,398 respondents reported using one or more prohibited substances or methods in the 12 months prior to survey administration. Specific doping prevalence rates for each individual substance / method categories ranged from 0.1% (for both diuretics / masking agents and stem cell / gene editing) to 4.2% for in-competition use of cannabinoids.

**Conclusion:**

Determining the prevalence of doping within different athlete populations is critical so that sport governing bodies can evaluate their anti-doping efforts and better tailor their programming. By measuring doping prevalence of specific categories of substances and methods, rather than just the overall prevalence of doping, this study also highlights where sport governing bodies should focus their future educational and detection efforts.

**Supplementary Information:**

The online version contains supplementary material available at 10.1186/s40798-024-00721-9.

## Key Points

Estimated doping prevalence among U.S. elite athletes ranged from 6.5 to 9.2%.

The most prevalent category of doping was in-competition use of cannabinoids (at 4.2%), whereas the least prevalent categories of doping were diuretics or masking agents (0.1%), stem cell or gene editing (0.1%), narcotics (0.2%), and hormone and metabolic modulators (0.2%).

Of the athletes who reported doping, most reported using only one substance or method.

**Supplementary Information:**

The online version contains supplementary material available at 10.1186/s40798-024-00721-9.

## Background

Anti-doping programs are sometimes criticized for being expensive and largely ineffective, while more sophisticated doping continues largely unabated [1]. Millions of dollars are spent annually by governments, sports organizations, and event organizers to fight doping globally [2] with an ambiguous return on investment. Some could argue that the very low number of anti-doping rule violations (ADRVs), a combination of both analytical (‘positive tests’) and non-analytical cases (‘investigations’), indicate that these efforts are successful. For example, according to data provided by the U.S. Anti-Doping Agency (USADA), in 2021, only 32 out of the 3,229 unique athletes who were tested, or about 1%, were sanctioned due to ADRVs. Yet the absence of a positive test does not equate to the absence of doping. Therefore, results from testing alone are unlikely to capture the full extent of doping among elite athletes in the United States (U.S.) competing at the Olympic and Paralympic level. Instead, more work is needed to estimate the prevalence of doping in this population.

Despite the importance of identifying how many elite athletes are doping^1^, the prevalence of doping among these athletes is not well-documented or well understood [3]. The primary problem appears to be that there is no gold standard method to estimate doping. For example, although drug and urine tests are critical for *detecting* and *deterring* doping, testing only captures a distinct moment in time [4]. Further, the World Anti-Doping Agency (WADA)’s Prohibited List contains hundreds of prohibited substances and methods, some for which there are no effective detection methods [5, 6]. Each drug on the Prohibited List has different windows of detection that further vary based on dozens of factors such as human physiology and pharmacology, detection sensitivity, and sample collection timing and frequency [5]. Therefore, other methods are needed to estimate doping prevalence beyond testing for the presence of a drug or its metabolite(s).

Doping prevalence studies, using different survey techniques, often are used as an alternative to testing to assess prevalence. The most common method to estimate prevalence is direct questioning [3] in which athletes self-report doping by answering questions that directly ask if they have used a substance or method. A recent review by the World Anti-Doping Agency (WADA) Working Group on Doping Prevalence found that the quality of direct questioning survey studies varies tremendously, but they offer several best practice recommendations that researchers can follow to improve the quality of their prevalence estimates [3]. To start, researchers should measure doping *behaviors* rather than infer prevalence from “proxy questions” (e.g., athlete’s intentions or willingness to dope). Second, survey questions should distinguish between doping and using non-prohibited substances (e.g., nutritional supplements, therapeutic medications). Third, researchers should use a clearly defined time frame, such as last 30 days or last 12 months, to allow for changes in doping behaviors and to better capture current rates of doping.

The goal of the current study was to estimate the prevalence of doping behavior in the previous 12 months among U.S. elite athletes (e.g., Olympians, Paralympians, Pan-American Games athletes) who are subject to drug testing under the World Anti-Doping Code. To our knowledge, no studies have estimated doping prevalence among such a large elite athlete population in the U.S. Consistent with the WADA Working Group’s recommendations, the current study asked separately about each substance / method category included in the WADA Prohibited List, rather than asking about doping more generally or only asking about a few specific categories of substances or methods. The current study also instructed athletes not to report use if they had a Therapeutic Use Exemption (TUE) for that substance or method so that athletes with medical conditions who used a substance or method for specific allowed purposes would not be counted as doping.

## Method

### Participants

Our sample frame consisted of all athletes subject to the USADA Protocol for Olympic and Paralympic Movement Testing (i.e., USADA anti-doping rules), a World Anti-Doping Code (“Code”)-compliant anti-doping program^2^. Athletes competing for most U.S. professional leagues (e.g., National Football League, National Basketball Association, Major League Baseball) are drug tested under collectively bargained employment agreements and are therefore not subject to the Code as these sports organizations are not Code signatories.^3^

Of the 2,616 athletes in the sampling frame, 1,595 athletes initially accessed the online survey via an emailed link. We excluded 13 athletes who did not provide consent, 63 athletes who indicated that they were younger than 18 years old (and thus ineligible for the study), 42 athletes who did not answer any items, 17 athletes who only answered the demographic items, and 4 athletes who did not answer any prevalence items. We also excluded 27 athletes who were missing responses to one or more prevalence items and did not report any use for the items they did answer, because there was not enough information to determine whether these athletes had doped (i.e., it is unknown whether their missing responses should be classified as “no use” or if they had purposely skipped the items so as to not report use but also not lie). By contrast, we retained two athletes in the sample who had some missing data on the prevalence items but who reported doping to one of the items that they did answer. Finally, we excluded an additional 31 athletes because they incorrectly answered two or more validation items (see description below), yielding a final analytic sample of 1,398 athletes (53.4% of the targeted population). Most participants had competed at the Olympics or Paralympics (45.4%) or World Championships (35.9%). The remaining 18.7% had competed at an international competition (e.g., Pan-American Games) or national championship.

### Procedures

#### Survey Administration

IRB approval was obtained from the Prevention Strategies IRB. Following IRB approval, the research team used a several step process to recruit athletes. First, to provide legitimacy for the study, USADA emailed all athletes in the sample to announce the study and inform them that they would be contacted by an independent research team inviting them to participate in an anonymous online survey. The email did not specifically mention that measuring doping was a goal of the study. Instead, the emails indicated that “Information from this survey will be used to better support you as an athlete.” USADA also announced the study to all the sport National Governing Bodies in case they received questions directly from their athletes. Then, the research team emailed all athletes to invite them to complete the survey and provided them with an anonymous, unique survey link through Qualtrics. Finally, the research team sent three follow-up reminders about the survey directly to non-respondents. Athletes were offered a $20 Amazon gift card code as an incentive for completing the survey; 86% of the final analytic sample provided information to receive the incentive. To protect athletes’ identities, athletes first completed the survey, then upon completion, if they chose to receive the incentive, they were given a link to another website, independent of the survey, where they could enter their email address to receive the incentive. Athletes accessed the survey between April 6 and June 1, 2020.

#### Direct Questioning Approach

Several steps were taken to encourage athletes to respond honestly. (1) Very few demographic questions were included on the survey, and prior to the section with the doping questions, instructions explicitly reminded athletes that there was no identifying information, including sport, attached to the survey, so neither researchers nor USADA could determine their identity. (2) Instructions emphasized that the goal of the research study was “to get a sense of what elite athletes, as a group, are doing, **not** to find out what you may have done or what substances you may have used.” (3) Each item about doping was presented on a separate screen, so athletes could quickly move on to the next item without their answer staying visible on the screen. Instructions informed athletes that this strategy was used to protect their privacy. (4) After USADA informed athletes about the study, all other contact came only from the research team. (5) All email contact reminded athletes that their answers would not be shared with USADA and that the research team would only report aggregate findings.

### Measures

The entire survey was about 15 minutes long and included measures of perfectionism, attitudes about doping, and normative beliefs about doping, in addition to the measures used in the current study, which are described in more detail below. The binary questions regarding doping were positioned very early in the survey after five demographic items and a seven-item perfectionism scale. The doping validation items (explained in greater detail below) were positioned toward the end of the survey.

#### Doping

WADA’s 2020 Prohibited List was used to develop 8 questions about whether athletes had use different categories of substances and methods without a TUE^4^ in the past 12 months. Given the time frame of the data collection, past 12-month use included use between April or May 2019 and April or May 2020. Each question provided common examples of doping substances or methods. For example, the first item asked: *During the ****past 12 months***, *have you used any prohibited ****anabolic agents? ****Examples of prohibited anabolic agents include but are not limited to: anabolic steroids like testosterone, DHEA, stanozolol, other anabolic agents like clenbuterol, selective androgen receptor modulators (SARMS like ostarine and LGD-4033.) If you ****only ****have used this substance while you had a Therapeutic Use Exemption (TUE) for this substance, select “No.”* The response options were 1 = No and 2 = Yes.

In addition, the survey included four questions asked about past 12-month use (without a TUE) of substances that are prohibited only in-competition. *In-competition* was defined according to the 2015 World Anti-Doping Code in force at the time of the survey (i.e., April – June, 2020). Specifically, the survey indicated “On the following screens, we are going to ask you about whether you have engaged in different behaviors **in-competition** over the past 12 months. **In-competition** includes the 12 hours before you were scheduled to compete through the end of the competition.” Each of the four items also included reference to in-competition (e.g., “In the **past 12 months**, have you used any prohibited **stimulants ***in competition*?) As with the items above, common examples were provided of the substance as part of each question, and respondents answered with a binary no/yes response scale.

#### Validation Items

To improve data quality, 5 validation items were interspersed among the doping questions that everyone would be expected to answer as “yes” (e.g., During the past 12 months, have you paid attention to your diet to ensure you were fueling properly?; Please answer “yes” to this question). Answering “no” to these items suggested careless responding [7]. 

#### Consistency Checks

A series of three questions were asked at the end of the survey about how often in the past 12 months athletes had used three of the substances: anabolic agents, asthma inhaler beyond allowable daily dose, and stimulants (in-competition), from 1 = *Never* to 4 = *Frequently.* Responses were compared against the no/yes use responses (e.g., someone who had not used an anabolic agent should say “no” to the first item and “never” to the frequency item).

#### Demographic Characteristics

*Age* was measured on a continuous scale based on the athlete’s reported age at the time of survey administration. *Sex* was measured by asking athletes to report whether they competed as a male or female in their sport. *Competition level* was measured by asking athletes to report the highest level of competition in their sport: (1) Olympics / Paralympics, (2) World Championship, (3) Other international competition (e.g., Pan-American or Para-Pan American Games, Youth Olympics), or (4) National, state or regional championship. Given low base rates in the latter two categories, these were collapsed together for analytic purposes. Athletes were not asked to report their sport so as to minimize concerns they could be identified. Instead, USADA provided the research team with contact email addresses separated by three risk categories: low, moderate, or high risk of doping using information about their sport, as is required in the WADA International Standard for Testing and Investigations. Specifically, USADA uses a proprietary formula that considers factors such as physiological risk (e.g., what are the physiological demands of the sport that might be affected by doping), historical risk (e.g., rates of anti-doping rule violations within a sport), and environmental risk (e.g., national sport prominence and financial opportunities) to stratify overall sport risk. Examples of a high-risk sport-discipline include road cycling or track and field events 3000 m or greater; moderate-risk sports include team sports such as basketball and ice hockey, and low-risk sports include badminton or golf^5^. These separate email lists enabled the research team to classify the athlete’s risk status in our analysis without compromising anonymity.

## Results

### Validity Check

Validation items were used to remove respondents who answered “no” to items where they should have responded “yes.” Such responses could reflect careless responding or potentially purposively deceptive responses (i.e., the athlete answered “no” to all items in the doping section without reading the items). A total of 218 athletes answered “no” to at least one validation item; 187 of these athletes answered “no” to one item, 26 answered “no” to 2 items, and 5 answered “no” to 3 items. The most common “no” responses were to the item asking whether they had paid attention to their diet to ensure that they were fueling properly during the past 12 months (*n* = 89), followed by the item about whether they had discussed their training program with their coach and / or athletics trainers in the past 12 months, asked only of athletes who had competed in the past year (*n* = 61), and the item about whether they had consumed at least 8 ounces (1 cup) of water each day during the past 12 months (*n* = 51). To allow for legitimate “no” responses (e.g., some athletes may not have paid attention to their diet in the past year; some athletes might have thought that the question to please answer “yes” was a trick question), we only excluded the 31 athletes who answered “no” to 2 or more of the validation items (∼ 2% of respondents). It might be tempting to assume that any athlete who incorrectly answered no to multiple items was lying or careless, but the responses to some of these athletes’ open-ended responses to the question “Is there anything else you would like us to know about doping in your sport” suggested otherwise. For example, one of the 31 excluded athletes noted, “I am constantly afraid of taking anything because I am worried that it will cause me to test positive. I am freaked out about advil, aleve, eye drops, and certain common flu medicine, especially antibiotics. My sport doesn’t even gain anything to help perform better from the banned substance list. However we have to follow the same rules as everyone else that risk having better performance because they are a more physical sport.” Another excluded athlete noted, “I wish that all medalist and top 8 (finalists) would all be tested in Para [sport redacted]. Instead they don’t test at all. It’s unreal.” These, and other, responses suggested that many excluded athletes provided thoughtful responses and that they themselves were unlikely to engage in doping. Nonetheless, because we cannot determine whether some athletes who said “no” to multiple validation items were careless or dishonest, we excluded all 31.

### Demographic Characteristics

The demographic characteristics of our analytic sample (compared to the full sample frame where there is available data) are provided in Table 1. There were no significant differences between the athletes in the analytic sample and the full population of USADA testing pool athletes in terms of sport-level risk category (*X*^2^ = 2.79, *p* = .248) or sex (*X*^2^ = 2.09, *p* = .148). The median age of the athletes in our analytic sample was 27 years old.

**Table 1:** Demographic characteristics of analytic sample

	Analytic Sample (*N* = 1,398)	USADA Testing Pool (Sampling Frame)
**Sex**		
Male	45.9%	52.1%
Female	53.9%	47.9%
Missing	0.2%	
**Highest Level of Competition**		
Olympic / Paralympic	45.4%	
World Championship	35.9%	
Other International (e.g., Pan-American)	16.0%	
National / state / regional championship	2.6%	
Missing	0.1%	
**Sport Risk Category**		
Low risk	55.6%	53.3%
Medium risk	10.0%	10.4%
High risk	34.5%	36.3%

### Individual Substance / Method Prevalence Results

Table 2 provides the percentage of athletes who reported using each substance / method. The first column lists the prohibited substance or method category. As noted in the column, some substance categories are prohibited at all times; in other words, both in and out of competition (e.g., anabolic agents), whereas others are only prohibited in competition (e.g., cannabinoids). The second column provides the percentage of athletes who responded “yes” to each binary item. Prevalence rates for each individual substance ranged from 0.1% (for both diuretics / masking agents and stem cell / gene editing) to 4.2% for in-competition use of cannabinoids. The third column provides the percentage of athletes who answered “rarely,” “occasionally,” or “frequently” for the three consistency check items.

The results in Table 2 are organized by whether they are substances that are always prohibited, substances that are prohibited in competition only, or prohibited methods. It is also possible to examine the results separately for categories of substances or methods that are considered as *non-specified* by WADA. Specifically, all anabolic agents, all peptide hormones, some hormone and metabolic modulators, most stimulates, and all methods except intravenous infusions are non-specified [23]. Typically, the primary reason such non-specified substances or methods would be used is to dope. By contrast, specified substances / methods are those that could have a non-doping reason for being used or present in a sample. Under this approach, 2.9% of athletes reported using a substance or method category that is either entirely or partially comprised of non-specified substances / methods.

**Table 2:** Percent of athletes who reported using each substance / method in the past 12 months

Substance	% Answered “Yes” to binary item	% Answered Rarely, Occasionally or Frequently to Consistency Check Item
**Prohibited substances (in and out of competition)**		
Anabolic agent^a^	1.1%	0.2%
Peptide hormones	0.4%	
Beta-2 agonists	0.3%	
Asthma inhaler beyond dose^b^	1.0%	2.6%
Hormone and metabolic modulators	0.2%	
Diuretics or masking agents	0.1%	
**Prohibited substances (in competition)** ^**d**^		
Stimulants^c^	0.8%	0.6%
Narcotics	0.2%	
Cannabinoids	4.2%	
Glucocorticoids	0.6%	
**Prohibited methods**		
Manipulate blood to improve performance / speed recovery	0.6%	
Stem cell or gene editing	0.1%	

NOTE: This table focuses on the percent of athletes reporting use of each substance/method. Because some respondents reported use of multiple substances/methods, the percentages are not additive

^a^1.2% of athletes reported use on either the binary or frequency item; 0.1% reported use on BOTH items

^b^2.6% of athletes reported use on either the binary or frequency item; 0.9% reported use on BOTH items

^c^0.9% of athletes reported use on either the binary or frequency item; 0.4% reported use on BOTH items

^d^Only athletes who reported competing in at least one competition over the past 12 months were included in these analyses. Therefore, the sample size for in-competition substance use was *n* = 1,321 for stimulants and *n* = 1,322 for the other three substances

### Overall Prevalence of Doping

Consistency check items were used to compute overall prevalence of doping in two ways. The first method classified anyone who reported “yes” and/or greater than never (i.e., rarely / occasionally / frequently) to one or more items as having doped. Using this method, 9.2% of athletes (*n* = 128) reported doping without a TUE in the past 12 months to one or more items from the Prohibited List. The second method classified athletes who reported “yes” *and* greater than never to those items that were asked about twice (e.g., the consistency check items) or “yes” to those items asked about only once as having doped. Using the latter method, 6.5% of athletes reported doping without at TUE in the past 12 months across all categories of the Prohibited List^6^. Notably, the calculated prevalence rates were identical if we included the 31 athletes who had answered two or more validation questions (i.e., 9.2% and 6.5% across the two calculation methods). Further, although it is tempting to assume that the higher prevalence number is automatically the better estimate (i.e., that everyone who reported use at any point had doped), the athletes’ open-ended responses suggested this was not necessarily true. For example, one athlete with inconsistent responses noted “Doping should be a life time band [sic]” and another athlete noted “I have never met anybody who has doped before. Nationally or internationally. I think it’s quite rare in [sport redacted].”

### Multiple Substance Prevalence Rates

Of the athletes who reported doping, the majority (84.4%) reported using only one substance or method (7.7% of the total sample). The remaining athletes reported that they used two (11.7%), three (2.3%), or four (1.6%) substances or methods. Table 3 shows the prevalence of each substance or method among athletes who reported use of only one substance or method (first two columns) compared to athletes who reported using two or more substances or methods (last two columns). Among athletes who reported only using one substance or method, the most common form of doping was in-competition use of Cannabinoids (41.3%). Among athletes who reported only using two or more substances or methods, the most common form of doping was also in-competition use of Cannabinoids (66.7%), although rates of in-competition use of stimulants (16.7–27.8%) and asthma inhalers beyond the allowable dose (30.0-40.0%) were also relatively common.

With the exceptions of Beta-2 agonists and stem cell / gene editing, the prevalence of each substance or method category was higher among athletes who reported using multiple substances / methods compared to those only reported use of one substance or method. For example, among the 108 athletes who reported using one substance or method in the past 12 months, 2.8% reported using peptide hormones, but among the 20 athletes who reported using 2 + substances or methods in the last 12 months, 15.0% reported using peptide hormones. One of the most noticeable differences between those who used only one substance / method compared to those who used two or more, was in-competition use: the prevalence of using substances that are prohibited in-competition was much higher among athletes who reported using 2 + substances or methods compared to athletes who reported using only one substance or method. For example, among athletes reporting use of one substance, 3.9–4.9% reported in-competition use of stimulants and 3.8% reported use of Glucocorticoids; however, among those reporting use of 2 + substances, 16.7–27.8% reported using stimulants and 22.2% reported using Glucocorticoids.

**Table 3:** Prevalence of each substance / method among athletes who reported use of 1 vs. 2 + substances

Substance	Reported Use of 1 Substance (*N* = 108)		Reported Use of 2 + Substances (*N* = 20)	
	% Answered “Yes” to binary item	% Answered Rarely, Occasionally or Frequently to Consistency Check Item	% Answered “Yes” to binary item	% Answered Rarely, Occasionally or Frequently to Consistency Check Item
**Prohibited substances (in and out of competition)**				
Anabolic agent^a^	11.1%	0.9%	15.0%	10.0%
Peptide hormones	2.8%		15.0%	
Beta-2 agonists	3.7%		0.0%	
Asthma inhaler beyond dose^b^	7.4%	25.2%	30.0%	40.0%
Hormone and metabolic modulators	1.9%		5.0%	
Diuretics or masking agents	0.0%		10.5%	
**Prohibited substances (in competition)**				
Stimulants^c^	4.9%	3.9%	27.8%	16.7%
Narcotics	0.0%		11.1%	
Cannabinoids	41.3%		66.7%	
Glucocorticoids	3.8%		22.2%	
**Prohibited methods**				
Manipulate blood to improve performance / speed/ recovery	3.7%		20.0%	
Stem cell or gene editing	0.9%		0.0%	

^a^Among athletes who used only one substance / method, 12.0% reported use on either the binary or frequency item; 0% reported use on BOTH items. Among athletes who used 2 + substances / methods, 20% reported use on either the binary or frequency item; 5% reported use on BOTH items

^b^Among athletes who used only one substance / method, 25.9% reported use on either the binary or frequency item; 6.5% reported use on BOTH items. Among athletes who used 2 + substances / methods, 45.0% reported use on either the binary or frequency item; 25.0% reported use on BOTH items

^c^Among athletes who used only one substance / method, 5.8% reported use on either the binary or frequency item; 2.9% reported use on BOTH items. Among athletes who used 2 + substances / methods, 33.3% reported use on either the binary or frequency item; 11.1% reported use on BOTH items

### Variation in Doping across Demographic Characteristics

To test whether mean age differed between those who reported doping and those who did not, independent sample t-test was used. Athletes who reported any doping were significantly younger (*M* = 26.4, *SD* = 6.2) than those who did not report doping (*M* = 28.1, *SD* = 6.9), *t* = 2.647, *p* = .008, *g* = 0.247. To test whether doping varied as a function of other demographic characteristics, a series of chi-square analyses was used. Given the relatively small size of those whose highest level of competition was a national, state, or regional championship (*n* = 37), these athletes were combined with other international competitions for our analyses. There were no significant differences in doping across these demographic groups (see Table 4)^7^.

**Table 4:** Doping prevalence as a function of demographic characteristics

Characteristic	% who reported any doping	*p*-value	Effect Size
**Sex**		0.058	ɸ = 0.051
Male	10.8%		
Female	7.8%		
**Highest Level of Competition**		0.814	ɸ = 0.017
National/International	9.2%		
World athlete	9.8%		
Olympic/Paralympic	8.7%		
**Risk category**		0.172	ɸ = 0.050
Low risk	8.1%		
Moderate risk	12.9%		
High risk	9.8%		

## Discussion

Depending on how inconsistent responses were counted, between 6.5% and 9.2% of U.S. elite athletes in our sample reported using one or more substances or methods from the WADA Prohibited List without a TUE in the past 12 months. These rates are much higher than the 1% of athletes who are sanctioned for anti-doping rule violations due to a positive sample, yet overall still quite low with the vast majority of participants not reporting any doping in the past 12 months. Notably, data collection for this study occurred from April 6 to June 1, 2020, at the start of the SARS-CoV-2 pandemic and associated lockdowns and competition cancelations. Because the prevalence items were asked retrospectively over the past 12 months, most of the window for potential doping occurred prior to the stoppage of athletic events. Further, two-thirds of the responding athletes completed the survey within the first week that the survey was open and an additional 18% completed it in its second week; thus, for most athletes, only a few weeks of the reporting period may have been impacted by these stoppages. We expect that few athletes who were not already doping would have started over those subsequent few weeks in the absence of the stoppages. However, future studies should keep this historical context in mind when comparing their findings to this study.

Importantly, there was some inconsistent reporting across the three validation items in the study. There can be multiple reasons for inconsistent responding across these items. First, we expect that it is harder for athletes who are lying to be consistent. Given our expectation that doping would be underreported, using multiple questions may have allowed us to “catch” some athletes in a lie. Second, some athletes may be more willing to admit doping when they are given a range of options (e.g., athletes may feel it is more socially acceptable to admit they have used an inhaler beyond the allowable dose a few times rather than admit “yes” to the binary item which they know will not distinguish them from someone who uses an inhaler beyond the allowable dose very often). Third, because the frequency validation items came at the end of the survey, without instructions reaffirming anonymity that preceded the original set of binary prevalence items, some athletes may have decided not to report doping on these later items. Yet it is likely that at least some inconsistent responses came from athletes who did not dope. For example, because the frequency validation items were at the end of the survey, athletes may not have read the questions as carefully, and they may have missed that the question about asthma inhaler use asked about use “beyond the allowable daily dose.” Inconsistent responses could also indicate speeding or misunderstanding; although, we tried to mitigate the former by removing athletes who failed our other validity check (i.e., answering more than 2 questions as “no” when they should have been yes), and we tried to mitigate the latter through cognitive interviews with several athletes conducted prior to launching the survey. Further evidence that some athletes who had inconsistent responses were either not doping, or at least not *knowingly* doping, came from their open-ended responses. Because it is impossible to determine whether inconsistent responses indicate lying vs. other problems, we calculated and reported prevalence using multiple methods.

### Implications

In general, the lowest rates of use were among the different types of non-specified substances or methods, such as peptide hormones, hormone and metabolic modulators, stem cell or gene editing, or blood manipulation, that are prohibited at all times. Overall, 2.9% of athletes reported any use of a non-specified substance or method. There are several potential explanations for the lower rates of non-specified substance / method. To start, one possibility is that athletes are more likely to comply with the anti-doping rules when the sanctions for using a particular substance or method are more punitive, as they are for non-specified substance / method use under the Code [9]. Specifically, given the inherent seriousness of non-specified substance or method use and the likelihood that these substances or methods were intentionally used for doping, athletes who test positive for a non-specified substance typically receive a sanction of four years of ineligibility; whereas athletes who test positive for a specified substance typically receive a sanction of two years, with the possibility of further reduction in the sanction. Another possibility is that rates are lowest among substances / methods that are more costly (e.g. gene doping) or those that require access to medical experts or equipment (e.g. blood transfusions). It is also possible that athletes are more aware that certain substances are not permitted, which could either lead athletes to be less likely to use them (vs. unintentional doping or using substances that they do not realize are prohibited) or to underreport use precisely *because* they know they are prohibited. Despite the overall lower rates of non-specified substance / method use, it is still important to note that 0.6% of athletes reported using blood manipulation, which speaks to the continued need of direct and indirect detection methods such as the athlete biological passport and investigations to identify hard to detect doping.

With regard to the use of specified substances, the highest rates were in-competition use of cannabinoids (4.2%) and use of asthma inhalers beyond the allowable daily dose (1%). Cannabinoids are not prohibited out-of-competition; therefore, athletes in the U.S. are more commonly using cannabis, marijuana, hashish and related products in oral and inhaled forms for recreation, recovery, and as sleep aids than ever before. This result could be partly due to increased availability and social acceptance. Athletes may perceive the in-competition detection of cannabinoids as low risk because carboxy-THC (the main metabolite of THC) has a urinary threshold of 150 ng/mL on the WADA Prohibited List, thus there is some chance of not testing positive after use. Further, the inclusion of the Substance of Abuse provision in the World Anti-Doping Code significantly reduced the length of Ineligibility sanctions from a potential two (or even four) years previously to three (or even one) month(s) today for athletes that can establish that the THC use occurred out-of-competition and was unrelated to sport performance. Therefore, athletes may be more willing to risk cannabis use because the penalties are comparatively low. The WADA category for asthma inhalers allows four specific inhaled beta-2 agonists (salbutamol, formoterol, salmeterol and vilanterol) to be used up to a daily dose limit without the need for a TUE. Our findings suggest that athletes may be using asthma inhalers for performance-enhancement purposes without a true therapeutic need, despite the evidence demonstrating that there is no performance benefit of beta-2 agonists in healthy individuals without a respiratory limiting medical condition [10, 11].

Whether doping varied across demographic characteristics also was examined. Doping varied by age—on average, athletes who reported doping were younger than those who did not report doping. This finding stands in contrast to anecdotal reports that older athletes are more likely to dope as they seek to extend their athletic careers or as they seek to return to play after an injury. Yet our finding is consistent with the general pattern of substance use, which increases from adolescence into young adulthood and then begins decreasing again [8]. The evidence about whether *doping* varies by age is less clear. A recent review of doping among younger athletes found no clear pattern about the relationship between age and doping across studies. Future studies should explore whether doping varies by age among other elite athlete populations in other countries.

By contrast, the prevalence of doping was not significantly different by sex or highest level of competition. The lack of sex differences stands in contrast with a review of doping among younger athletes (ages 10–21) [12] and a review of doping behaviors more generally [13], which both found higher rates of doping among male athletes than female athletes. Notably, however, few studies have examined gender differences among young adult elite athletes. Indeed, in their recent systematic review of 105 studies of doping prevalence in competitive sport, Gleaves et al. [3] concluded that no meaningful conclusions could be made about gender differences in doping, given that so few studies examine prevalence by gender. Further, consistent with our results, a recent study of Danish elite adolescent and young adult athletes found no gender differences in doping [14]. Therefore, although younger male athletes may engage in higher rates of doping, this pattern may disappear among older, elite athletes. Future studies should continue to measure and test gender or sex differences in doping prevalence.

Perhaps surprisingly, prevalence of doping also did not vary by risk level of the sport (as defined by USADA). One possibility is that these risk levels are used to determine anti-doping requirements placed on athletes within each risk group. For example, athletes in the highest risk group typically receive the highest frequency of urine and blood testing throughout the year, are required to provide a 1-hour window of availability every single day of the year where they must be available for testing (although not necessarily tested during this 1 h) or they are subject to strict missed test and whereabouts failure violations. The lack of differences across sport risk category could suggest that detailed risk-based testing strategies, as opposed to simply random testing, combined with tailored athlete whereabouts and education requirements have been successful at mitigating doping risk across the athlete population.

### Study Limitations

The findings should be considered within the context of several limitations. First, all studies that rely on self-reports about doping require athletes to be honest about engaging in dishonest behavior and thus are likely to suffer from some degree of underreporting. Athletes may underreport doping due to concerns about negative consequences if they admit to doping (e.g., social stigma; being banned from sport) [3] or due to social desirability bias more generally [15–17]. Importantly, however, many studies examining sensitive topics have found that self-report data can be valid, especially when specific steps are used to encourage honest responding [18]. Our study used multiple strategies to promote honest responding. For example, our study used anonymous responding, used a third party to collect the data to reassure athletes of the anonymity of their responses, collected limited demographic data and explicitly let athletes know that we were doing this to protect their identity, and only asked one sensitive question per screen so that any affirmative answers were on the screen for a limited time (see method section for more details).

A second limitation is that non-response bias may also have contributed to underestimation of doping prevalence if athletes who dope were less likely to complete the survey. To encourage survey participation, our recruitment emails used broad language so as not to discourage athletes who dope from responding. Importantly, however, more than one-half of eligible athletes completed the survey, and there were no differences in completion rates across risk level of the sport, suggesting that athletes from high-risk sports were just as likely to complete the survey as those from low-risk sports. Further, only about 3% of athletes did not answer the prevalence items once they opened the survey, indicating that very few athletes opted out of the survey once they began it. Controls for decreasing social desirability bias, guaranteeing confidentiality, improving respondent cooperation, and procuring reliable responses were all built into the survey design, implementation, and analysis process.

A third limitation of the current study is that a limited number of demographic characteristics were measured; characteristics, such as sport, that are likely linked to doping prevalence, were not measured. As noted above, demographic characteristics that were included in the survey were purposely limited to protect athletes’ identity and promote honest responding. The tradeoff, however, is that less information available for determining who is at the greatest risk of doping.

Given the limitations of direct questioning, researchers have developed indirect estimation methods such as randomized response technique (RRT) [19] to estimate prevalence [14, 20]. In RRT, the respondent is instructed to answer questions based on probability. For example, in an RRT survey, respondents may be asked to flip a coin or choose a random number, and based on the outcome, they should answer honestly or dishonestly. These veiled survey protocols are intended to encourage honest responses yet there is evidence that many respondents either do not trust the procedures [21] or are confused by the task when completed as a self-administered survey rather than as part of an in-person or phone interview [21]. In addition, indirect estimation techniques may not be ideal when researchers want to conduct a detailed examination of many illicit behaviors, such as each category on the World Anti-Doping Association (WADA) Prohibited List. Although possible, using a method such as RRT would require respondents to administer the probability-based instructional trigger before each item, substantially increasing respondent burden and increasing the risk of obtaining incomplete, unreliable, or invalid results.

### Future Directions

First, although some work has attempted to identify predictors of doping among younger athletes [12] and of doping behavior more broadly [13], more work is needed to identify which modifiable risk and protective factors are associated with doping among elite athletes competing at the highest levels. Indeed, a meta-analysis of studies trying to identify predictors of doping among elite athletes [22] identified very few studies that focused on doping *behavior* (as opposed to intentions or susceptibility to doping), underscoring the need for more work in this area. Such knowledge would allow prevention efforts to better address these predictors. Further, future studies should test whether predictors vary across types of substances / methods. Second, we echo calls by Gleaves et al. [3] to use best practices for collecting prevalence estimates. Using similar methodology across studies will allow for better comparisons across populations and over time. Third, we argue that future studies should specifically test different methodological approaches and evaluate the accuracy of estimates under each approach. Fourth, future studies should repeat the current doping prevalence study over time to understand if anti-doping program changes are positively impacting the clean sport behavior of athletes. Finally, future studies should focus on identifying athletes who have been *deterred* from doping by effective anti-doping programs, in addition to estimating the prevalence of doping. In fact, anti-doping programs are caught in a paradoxical situation by which if 100% effectiveness is measured by 100% deterrence, then no athlete will be caught doping. Conversely, if an anti-doping detection program is 100% ineffective, the same answer is obtained -  no athlete will be caught doping. Determining whether the gap between the detection rate and self-reported prevalence rate narrows or widens over time can serve as a standard of success of doping deterrence measures.

## Conclusion

As noted in the introduction, there is no gold standard method for estimating prevalence. Biological testing only captures a distinct moment in time and cannot detect all substances and methods on WADA’s Prohibited List. Surveys that use direct questioning, as this study did, are subject to challenges with deliberate underreporting, whereas surveys that use indirect questioning are only effective if athletes understand and trust the directions. Further, indirect questioning can increase participant burden when assessing many illicit behaviors within the context of a larger survey. Therefore, efforts to capture true prevalence of doping require continued triangulation of all three types of studies. Our study contributes to those efforts by being the first study, to our knowledge, to assess doping prevalence among elite athletes, subject to the WADA code, in the United States using the complete list of categories from the WADA Prohibited List. Depending on how the consistency check items were treated, estimates of use without a TUE among our sample of elite U.S. athletes in the past 12 months across all Prohibited List categories ranged from 6.5 to 9.2%. Further, the survey found that a smaller percentage of athletes, 2.9%, reported using non-specified substances or methods, the most egregious type of doping. In other words, our results suggest that most U.S. elite athletes competing at the international or Olympic / Paralympic level are not doping. Future studies should compare doping among other similar elite athlete populations, as well as examine predictors of doping, to determine what combination of factors (e.g., norms, attitudes, education, testing, deterrence strategies, policies) may differentially affect doping across countries.

### Electronic Supplementary Material

Below is the link to the electronic supplementary material.

Supplementary Material 1

## Data Availability

We plan to share the dataset of the items used for this study via the UNCG NC DOCKS Dataverse, which is hosted by the UNC Dataverse at the Odum Institute, University of North Carolina at Chapel Hill. NC DOCKS Dataverse is the institutional data repository of UNC Greensboro and data are made publicly available under a CC0 Public Domain Dedication Waiver. This data repository adheres to the FAIR standards and guidelines created by the Data-PASS, including a commitment to long-term preservation.

## Footnotes

1 For the purposes of this paper, we define doping as the use of substances or methods prohibited under the WADA Prohibited List.

2 All athletes in a USADA testing pool (either the Registered Testing Pool or the Clean Athlete Program) are elite level athletes subject to the USADA Protocol for Olympic and Paralympic Movement Testing and were included in our sampling frame. All survey respondents have received formal education across the diverse categories of the substances and methods included on the WADA Prohibited List.

3 Professional athletes who compete for the U.S. in an event subject to the Code (e.g., the Olympic games), *are* subject to a Code compliant anti-doping program from their National Anti-Doping Organization (e.g., USADA, International Federation, or Major Event Organizer) during the time they are representing the U.S.

4 TUE: A Therapeutic Use Exemption allows an athlete with a medical condition permission to use a Prohibited Substance or Prohibited Method for therapeutic purposes.

5 Specific details of which sports are in each risk category are not provided to protect USADA’s individual sport testing plans.

6 The prevalence estimates of 6.5–9.2% in the study are generally consistent with athletes’ perceptions of doping by other athletes within their sport. Specifically, another question on the survey asked athletes what percent of U.S. athletes in their sport are doping and the reported mean for this item was 11.2%. Although normative estimates tend to overestimate behaviors, there is evidence that people are more accurate at estimating the behavior of more proximal reference groups [1].

7 All reported analyses used any doping reported on either the binary *or* frequency items. We repeated the analyses comparing those who consistently reported doping to *both* items and found the same results; athletes who reported doping were significantly younger (*M* = 25.9) than those who did not report doping (*M* = 28.0), *t* = 2.888, *p* = .004. There were no significant differences across the other three demographic groups.

## Abbreviations

ADRV: Anti-Doping Rule Violation
RRT: Randomized Response Technique
SARMS: Selective Androgen Receptor Modulators
TUE: Therapeutic Use Exemption
USADA: United States Anti-Doping Agency
WADA: World Anti-Doping Agency
