# Supplementary material for: Doping Prevalence among U.S. Elite Athletes Subject to Drug Testing under the World Anti-Doping Code
Source: Sports Med Open. 2024 May 20;10:57. doi: 10.1186/s40798-024-00721-9 (PMC11102888; doi:10.1186/s40798-024-00721-9)
Supplement: Supplementary file 1 — Supplementary Material 1 [file 40798_2024_721_MOESM1_ESM.pdf]

Doping prevalence among U.S. elite athletes subject to drug testing under the World Anti-Doping Code

1. Ann Kearns Davoren*, 2. Kelly Rulison, 3. Jeff Milroy, 4. Pauline Grist, 5. Matthew Fedoruk, 6. Laura Lewis, 7. David Wyrick

1. Prevention Strategies, Greensboro, NC, USA
2. Prevention Strategies, Greensboro, NC, USA
3. The University of North Carolina Greensboro, Greensboro, NC, USA
4. The University of North Carolina Greensboro, Greensboro, NC, USA
5. U.S. Anti-Doping Agency (USADA), Colorado Springs, CO, USA
6. US Anti-Doping Agency (USADA), Colorado Springs, CO, USA
7. The University of North Carolina Greensboro, Greensboro, NC, USA

*akdavoren@preventionstrategies.com

**Prevalence Items Used in this Manuscript**

***Binary prevalence items with preceding instructions and disclaimer (all with response scale of No, Yes):***

On the following screens, we are going to ask you about whether you have engaged in different behaviors over the past 12 months.

As a reminder, **we take your anonymity very seriously** and have taken multiple measures to safeguard your responses.

To protect your privacy as you take the survey, each question about your behavior will be asked on a different page so that your answers will only be on the screen briefly.

The goal of this research study is to get a sense of what elite athletes, as a group**,** are doing, **not** to find out what you may have done or what substances you may have used. Therefore, **your honesty is extremely important** and will help us understand elite athletes like you so that you can be better supported in your sport.

As a reminder, there is **no identifying information attached to this survey**, including sport, so there is no way for researchers or USADA to identify who you are.

- During the **past 12 months**, have you used any prohibited **anabolic agents?**

  Examples of prohibited **anabolic agents** include, but are not limited to: anabolic steroids like testosterone, DHEA, stanozolol, other anabolic agents like clenbuterol, selective androgen receptor modulators (SARMs) like ostarine and LGD-4033

  *If you **only** have used this substance while you had a Therapeutic Use Exemption (TUE) for this substance, select "No."*

- During the **past 12 months**, have you used any prohibited **peptide hormones or growth factors**?

  Examples of prohibited **peptide hormones or growth factors** include, but are not limited to: erythropoietin (EPO), peptides, human growth hormone (hGH)

  *If you only have used this substance while you had a Therapeutic Use Exemption (TUE) for this substance, select "No."*

- During the **past 12 months**, have you used any prohibited **hormone and metabolic modulators?**

  Examples of prohibited hormone and metabolic modulators include, but are not limited to: insulin, clomiphene, tamoxifen, anastrozole, aromatase inhibitors, GW1516, meldonium

  *If you **only** have used this substance while you had a Therapeutic Use Exemption (TUE) for this substance, select "No." All hormonal contraceptives are allowed.*

- During the **past 12 months**, have you used any prohibited **diuretics or masking agents?**

  Examples of prohibited **diuretics** include, but not limited to: hydrochlorothiazide, spironolactone, plasma expanders

*If you **only** have used this substance while you had a Therapeutic Use Exemption (TUE) for this substance, select "No."*

- During the past **12 months**, have you used any prohibited **substances or methods to manipulate your blood to improve your performance or speed recovery?**

  These substances and methods include, but are not limited to: intravenous infusions (IVs), blood boosters, blood transfusions

  *If you **only** have used this substance or method while you had a Therapeutic Use Exemption (TUE) for this substance or method, select "No."*

- During the past **12 months**, have you used any prohibited **stem cell or gene editing agents with the goal of enhancing performance** (e.g., use of gene doping agents)?

  *If you **only** have used this substance while you had a Therapeutic Use Exemption (TUE) for this substance, select "No."*

- In the **past 12 months**, have you used any prohibited **stimulants** *in competition*?

  Examples of prohibited stimulants include, but are not limited to: amphetamine, methylphenidate pseudoephedrine, cocaine

  *If you only have used this substance while you had a Therapeutic Use Exemption (TUE) for this substance, select "No."*

- In the past **12 months**, have you used any prohibited **narcotics** *in competition*?

  Examples of prohibited **narcotics** include, but are not limited to: oxycodone, morphine, methadone hydromorphone.

  *If you only have used this substance while you had a Therapeutic Use Exemption (TUE) for this substance, select "No."*

- In the **past 12 months**, have you used any prohibited **cannabinoids** *in competition*?

  Examples of prohibited cannabinoids include, but are not limited to: cannabis, marijuana, hashish, synthetic cannabinoids

  *If you only have used this substance while you had a Therapeutic Use Exemption (TUE) for this substance, select "No."*

- In the **past 12 months**, have you used any prohibited **glucocorticoids** *in competition* **by oral, intramuscular, intravenous, or rectal modes of administration**?

  Examples of prohibited glucocorticoids include, but are not limited to: prednisone, cortisone, dexamethasone, triamcinolone

  *If you only have used this substance while you had a Therapeutic Use Exemption (TUE) for this substance, select "No."*

***Frequency prevalence items (all with response scale of Never, Rarely, Occasionally, Frequently):***

- During the **past 12 months**, how often have you used any prohibited **anabolic agents?**

Examples of prohibited anabolic agents include, but are not limited to: anabolic steroids like testosterone, DHEA, stanozolol, other anabolic agents like clenbuterol, selective androgen receptor modulators (SARMs) like ostarine and LGD-4033

*If you only have used this substance while you had a Therapeutic Use Exemption (TUE) for this substance, select "Never."*

- During the **past 12 months**, have you used permitted asthma inhalers (e.g., albuterol, formoterol, salmeterol) **beyond the allowable daily dose**?

- During the **past 12 months**, how often have you used any prohibited **stimulants *in competition***?

  Examples of prohibited stimulants include, but are not limited to: amphetamine, methylphenidate pseudoephedrine  cocaine

  *If you only have used this substance while you had a Therapeutic Use Exemption (TUE) for this substance, select "Never."*
